# Loss of oncogenic miR-155 in tumor cells promotes tumor growth by enhancing C/EBP-β-mediated MDSC infiltration

**DOI:** 10.18632/oncotarget.7150

**Published:** 2016-02-03

**Authors:** Sinae Kim, Jin Hoi Song, Seokho Kim, Peng Qu, Betty K. Martin, Waheed S. Sehareen, Diana C. Haines, Pengnian C. Lin, Shyam K. Sharan, Suhwan Chang

**Affiliations:** ^1^ Department of Biomedical Sciences, Department of Physiology, University of Ulsan School of Medicine, Seoul, South Korea; ^2^ Aging Research Center, Korea Research Institute of Bioscience & Biotechnology, Taejeon, South Korea; ^3^ Pathology Histotechnology Laboratory, Leidos Inc., Frederick National Laboratory for Cancer, Frederick, MD, USA; ^4^ Mouse Cancer Genetics Program, National Cancer Institute, Frederick, MD, USA

**Keywords:** miR-155, MDSC, C/EBP-beta, cytokine, breast cancer

## Abstract

The oncogenic role of microRNA-155 (miR-155) in leukemia is well established but its role in other cancers, especially breast cancer, is gradually emerging. In this study we examined the effect of mir-155 loss in a well-characterized spontaneous breast cancer mouse model where *Brca1* and *Trp53* are deleted by *K14-Cre.* miR-155 is known to be up-regulated in BRCA1-deficient tumors. Surprisingly, complete loss of miR-155 (*miR-155^ko/ko^*) did not alter the tumor free survival of the mutant mice. However, we found increased infiltration of myeloid derived suppressor cells (MDSCs) in miR-155 deficient tumors. In addition, cytokine/chemokine array analysis revealed altered level of cytokines that are implicated in the recruitment of MDSCs. Mechanistically, we identified C/EBP-β, a known miR-155 target, to regulate the expression of these cytokines in the miR-155-deficient cells. Furthermore, using an allograft model, we showed that inhibition of miR-155 in cancer cells suppressed in vivo growth, which was restored by the loss of miR-155 in the microenvironment. Taken together, we have uncovered a novel tumor suppressive function of miR-155 in the tumor microenvironment, which is also dependent on miR-155 expression in the tumor cells. Because of the oncogenic as well as tumor suppressive roles of miR-155, our findings warrant caution against a systemic inhibition of miR-155 for anticancer therapy.

## INTRODUCTION

miR-155 is a well-known oncogenic microRNA encoded by the *BIC (B-Cell Insertion cluster)* gene [1]. *BIC* transcribes a 1.5kb noncoding RNA, which is processed to generate mature miR-155[2]. As the name suggests, the BIC locus was identified as a proviral insertion site causing B-cell lymphoma [1]. Subsequently, miR-155 was identified in the transcript and shown to be responsible for the oncogenic nature of this locus [2]. Many studies have shown that miR-155 is up-regulated in multiple types of cancer including lymphoid, breast, colon, lung and pancreas [3–5]. A number of studies have been undertaken to identify the targets of miR-155 [6, 7]. There are at least 150 validated targets (from MirTar Base) of miR-155. More recently, an Ago-CLIP-Seq study using WT or miR-155-deficient T-cells revealed 250 targets [7]. Although most of these new targets remain to be validated, the existence of such many potential targets suggests that miR-155 has multiple functions that might be dependent on cellular context.

Functional studies using knockout mouse models have revealed that miR-155 is involved in B and T-cell development as well as germinal center formation [8, 9]. Subsequent studies have provided evidence to support a role for miRNA in normal immune system development and function [10, 11]. It is induced upon the activation of B or T cells and controls inflammatory response by functioning in monocytes, macrophage and dendritic cells [12]. Its role in the tumor microenvironment has also been studied. A study using EL4 and B16F10 melanoma cell lines in a allograft model reported that the absence of miR-155 in recipient mice resulted in faster tumor growth [13]. In this study, miR-155 found to modulate IFN gamma production in T cells. Another study revealed that miR-155 knockdown in myeloid cells accelerated tumor growth [14]. miR-155 is also shown to be required for tumor associated macrophage (TAM) mediated antitumor function [14, 15].

In contrast to TAM that negatively regulates tumor growth, there are a number of suppressor cells that inhibits normal antitumor immune activity. Among them, the myeloid derived suppressor cells (MDSC) are known to be regulated by miR-155 via SHIP-1 [16]. In a line with this finding, MDSCs was shown to require miR-155 to facilitate tumor growth [17]. However, the latter report showed the loss of host miR-155 overall promoted antitumor activity, which is not consistent with the results of other studies. Interestingly, another recent study revealed the loss of miR-155 in MDSCs enhanced its recruitment and function in solid tumor [18], suggesting that the role of miR-155 may vary depending on the tumor type and the model system.

To address some of the inconsistencies in previous studies and dissect the diverse role of miR-155 in breast cancer, we have examined its function in two different contexts. First, we have utilized a well-characterized *Brca1^cko/cko^;Trp53^cko/cko^; K14-Cre* mouse model because miR-155 is upregulated in BRCA1-deficient tumors [19]. We have examined the effect of miR-155 loss on tumorigenesis by germ-line inactivation of miR-155. In these mice, miR-155 is deficient in both the tumor as well as the tumor microenvironment. Second, we have used an allograft model to examine the growth of LLC1 cells with stable knockdown of miR-155 in *miR-155^ko/+^* or *miR-155^ko/ko^* mice. Our results suggest a dual role for miR-155 in the tumor and the tumor microenvironment. The implications of our findings on the use of miR-155 as a therapeutic target are discussed.

## RESULTS

### Germ-line inactivation of miR-155 does not affect the tumor free survival of Brca1/Trp53 mutant mice

**Figure 1:**
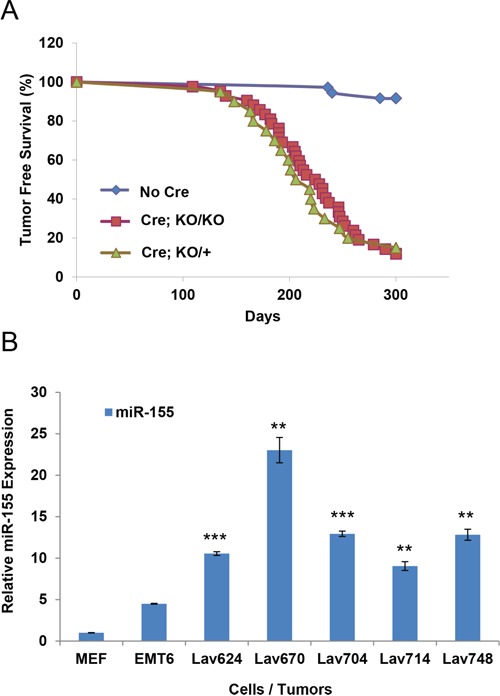
Effect of miR-155 on the tumor free survival of Brca1^cko/cko^;Trp53^cko/cko^;K14-Cre mice **A.** Tumor free survival of *Brca1^cko/cko^;Trp53^cko/cko^;K14-Cre* on *miR-155^ko/+^* (Cre; KO/+) or *miR-155^ko/ko^* (Cre; KO/KO) genetic backgrounds. Mice without *K-14 Cre* (No Cre) were used as control. **B.** Real-time PCR-based quantification of miR-155 in *Brca1^cko/cko^;Trp53^cko/cko^;K14-Cre* tumors. Lav624, 670, 704, 714 and 748 cells are from miR-155 KO/+ tumors. MEF (Mouse embryonic fibroblast) and EMT6 (mouse mammary tumor cell line with WT BRCA1) were used as controls. **p<0.01, ***p<0.001 for all panels. Representative result of three experiments.

Considering the oncogenic role of miR-155 and its up-regulation in *BRCA1-*deficient tumors [20] we reasoned its inactivation might increase the tumor latency or reduce the tumor incidence. To examine the possibility, we crossed *miR-155^ko/ko^* mice with well established *Brca1^cko/cko^;Trp53^cko/cko^;K14-Cre* mouse model and monitored a cohort of *Brca1^cko/cko^;Trp53^cko/cko^;K14-Cre*; *miR-155^ko/ko^* and *Brca1^cko/cko^;Trp53^cko/cko^;K14-Cre*; *miR-155^ko/+^* mice for tumor incidence [21]. Surprisingly, we found the tumor free survival of *Brca1^cko/cko^;Trp53^cko/cko^;K14-Cre* mice was not significantly changed in *miR-155^ko/ko^* and *miR-155^+/ko^* genetic backgrounds (Figure 1A). In addition, we did not find any significant histological differences between the tumors from the two groups (Supplementary Figure 1A, Supplementary Table 1). Consistent with our previous findings, we observed a significant up-regulation of miR-155 in tumors from *Brca1^cko/cko^;Trp53^cko/cko^;K14-Cre* mice compared to EMT6 mammary cancer cells and primary mouse embryonic fibroblasts (MEF) (Figure 1B). These results suggest that germline inactivation of miR-155 does not affect the tumor free survival of *Brca1^cko/cko^;Trp53^cko/cko^;K14-Cre* mice. However, considering the known oncogenic role of miR-155 in breast cancer cell as well as its tumor suppressive effects via normal immune activation[14, 22], it is possible that the two opposing effects might compensate each other.

### Increased MDSC infiltration in miR-155-deficient tumor microenvironment

**Figure 2:**
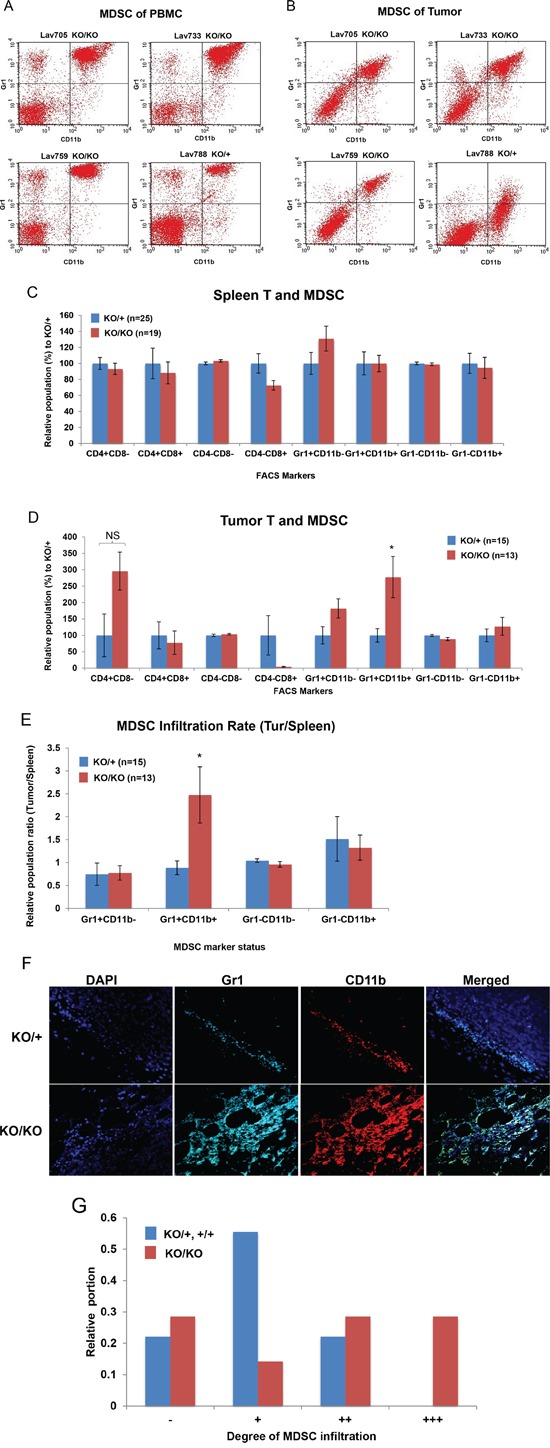
Increased MDSC infiltration in miR-155-deficient tumors **A, B.** Representative data of FACS analysis of PBMC (A) and tumor (B). Cells in the right upper quadrant indicate MDSC population. Lav705, 733 and 759 are from miR-155^ko/ko^ (KO/KO), and Lav788 from miR-155^ko/+^ (KO/+) mouse. **C.** CD4/CD8 positive T cells and MDSC cell analysis in Spleen obtained from KO/+ or KO/KO tumor bearing mouse (n=44). Relative population of each type of cells in KO/KO spleen was expressed as percentage of the KO/+. **D.** CD4/CD8 positive T cells and MDSC cell analysis in tumor obtained from KO/+ or KO/KO tumor bearing mice (n=28). The same analysis in C was done with the cells from mammary tumor. Note a significant increase of Gr1/CD11b double positive, MDSC in KO/KO tumor. **E.** MDSC infiltration ratio calculated by Tumor MDSC portion divided by spleen MDSC. **F.** Representative picture of Immunofluorescence staining of MDSC infiltrated in miR-155^ko/+^ (KO/+) and miR-155^ko/ko^ (KO/KO) tumors, using Gr1 and CD11b antibodies. **G.** Quantification of MDSC infiltration based on immunofluoresence analysis. Depending on the MDSC infiltration, the tumors were scored blindly from – to +++. Red/blue bar represent the portion of each group from miR-155^ko/+^ (KO/+, blue) or miR-155^ko/ko^ (KO/KO, red) mammary tumors. * p<0.05.

Loss of miR-155 in MDSCs is reported to exert increased migration and immune suppressive potential [17]. Therefore, we first examined whether MDSCs play a role in tumorigenesis in *Brca1^cko/cko^;Trp53^cko/cko^;K14 Cre*; *miR-155^ko/ko^* mice as well. To measure the infiltrated MDSCs in mammary tumor, we used two known MDSC markers, CD11b and Gr1. FACS analysis using these two antibodies labeled with FITC or PE showed a double positive population that is enriched in the tumor and peripheral blood from the tumor-bearing mouse (Figure 2A and 2B). Using this approach, we compared the number of infiltrated MDSCs in the spleen (n=44) and tumors (n=28) of mutant and control mice. We also examined CD4 and CD8 positive cells in these samples. In the spleen, we observed no difference in the number of MDSCs between the two genotypes, suggesting that the generation of MDSCs in the tumor bearing mice is not affected by the presence or absence of miR-155 (Figure 2C). We did not observe any significant difference in the CD4 and CD8 positive cells. In contrast, we found an increase in the MDSC population in the tumors from *miR-155^ko/ko^* mice (Figure 2D). We also observed increased CD4 positive and decreased CD8 positive cells in the *miR-155^ko/ko^* tumors but the differences were not significant due to high variability observed in the tumors. When the infiltration rate was calculated by dividing the portion of MDSC in tumor by the portion of MDSC in spleen, we observed a significant increase in the ratio in *miR-155^ko/ko^* mice (Figure 2E). This was further confirmed by visualizing MDSCs in the tumors by IHC staining. As shown in Figure 2F, the degree of MDSC infiltration in the peripheral region of tumor detected by CD11b and GR1 antibodies, greatly varies between samples. However, when we scored the degree of the infiltration using an arbitrary four tier grading system (−, +, ++ and +++), we observed that the tumors from *miR-155^ko/ko^* mice have higher degree of the MDSC infiltration (Figure 2G, Table 1). Based on these observations, we concluded that the MDSCs are more efficiently recruited to the tumor sites in the *miR-155^ko/ko^* mice. It is possible that this contributes to the tumor growth in miR-155-deficient background (Figure 1A). These findings are consistent with other reports showing increased MDSC infiltration in the absence of miR-155 [18].

**Table 1:** Quantification of MDSC infiltration using an arbitrary four tier grading system (−, +, ++ and +++)

Tumor	miR-155	CD11b	GR1
LAV271	+/+	++	++
LAV443	KO/+	−	−
LAV792	KO/+	+	+
LAV755	KO/+	++	++
LAV790	KO/+	+	+
LAV284	KO/+	+	+
LAV670	KO/+	+	+
LAV707	KO/+	−	−
LAV173	KO/+	+	+
LAV379	KO/KO	+	+
LAV417	KO/KO	+	+
LAV590	KO/KO	−	−
LAV614	KO/KO	−	−
LAV736-2	KO/KO	++	++
LAV736	KO/KO	+++	+++
LAV761	KO/KO	++	++
LAV713	KO/KO	+++	+++
LAV773	KO/KO	++	++
LAV162	KO/KO	−	−
LAV314	KO/KO	−	−
LAV697	KO/KO	++	++
LAV281	KO/KO	+++	+++
LAV352	KO/KO	+++	+++

### Cytokine array analysis revealed dynamic changes in cytokine/chemokine expression in the absence of miR-155

**Figure 3:**
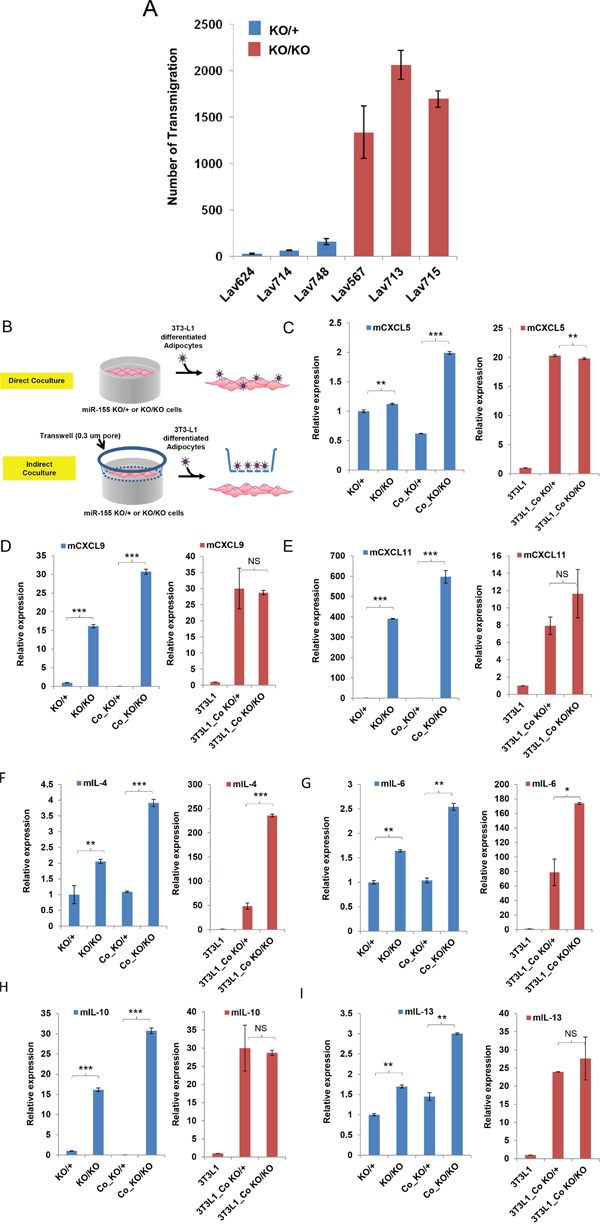
Loss of miR-155 stimulates MDSC migration and up-regulates cytokine/chemokine expressions **A.** Migration assay of MDSC cells treated with culture media from miR-155^ko/+^ (KO/+) or miR-155^ko/ko^ (KO/KO) breast cancer cells. Bars 1-3 represent breast cancer cells that are miR-155^ko/+^ (KO/+) and 4-6 represent miR-155^ko/ko^ (KO/KO) cells. Lav624, 714, and 748 cells are from KO/+, and Lav567, 713, and 715 cells are from KO/KO tumors. **B.** Schematic diagram showing cancer cell/adipocyte co-culture. For indirect co-culture, transwell was applied. **C-E.** Real time PCR results of the chemokines that are pre-screened from cytokine/chemokine array. Co- indicates co-culture C:CXCL5, D:CXCL9, E:CXCL11. **(F-I) Real time PCR results of cytokines.** F: IL-4, G: IL-6, H: IL-10, I: IL-13. *p<0.05, **p<0.01, ***p<0.001 for all panels. Representative result of two (A) or three experiments (C-I).

We next examined whether miR-155-deficient tumor cells are better adapted to attracting MDSCs than the tumor cells expressing miR-155. Indeed, an *in vitro* migration experiment using normal MDSC and conditioned medium from *miR-155^ko/ko^* and *miR-155^+/ko^*. Tumor cells showed that the *miR-155^ko/ko^* conditioned medium induced much more MDSC migration than *miR-155^+/ko^* (Figure 3A). This suggested that some unknown tumor derived factor(s) from *miR-155^ko/ko^* tumor cells were attracting more MDSCs. To identify such factor(s), we designed a co-culture system consisting of tumor cells plus differentiated 3T3-L1 cells [23] (Figure 3B, Supplementary Figure 2). Using this system, we aimed to mimic the known interplay between breast tumor and adipose cells, which produce high levels of cytokines/chemokines, in vitro. We confirmed that indirect co-culture system was functioning properly, by measuring the expression of a number of cytokines that are known to be up-regulated [24] (Supplementary Figure 3). Using this indirect co-culture system and a PCR array, we examined the cytokine/chemokine expression changes in 3T3-L1 derived-adipocytes and *miR-155^ko/ko^* and *miR-155^+/ko^* tumor cells separately. We identified a number of tumor-driven cytokines/chemokines that are differentially regulated in the absence of miR-155 (Table 2). These included CXCL5, 9, 11 as well as IL-4, 6,10,13, which are up-regulated and known to be involved in the MDSC migration [25, 26]. Real time PCR analysis confirmed up-regulation of these factors (Figure 3C–3I) in co-cultured *miR-155^ko/ko^* cells.

**Table 2:** List of up-or down-regulated cytokine/chemokine genes obtained from cytokine PCR array

Position	Symbol	Fold Regulation
C11	Cxcl9	222.8609
E05	Il1rn	115.3601
A07	Ccl11	58.0812
C02	Cx3cl1	45.5696
A09	Ccl17	39.9466
G08	Tnfsf10	26.5382
E02	Il18	21.5557
B01	Ccl22	12.5533
C05	Cxcl11	12.295
D10	Il15	12.2101
F04	Il7	11.7942
D07	Il12a	11.3924
G09	Tnfsf11	7.7812
B06	Ccl7	7.6741
G04	Tgfb2	7.1602
A11	Ccl2	4.4383
D03	Ifna2	4.3772
B10	Csf1	4.1989
D05	Il10	3.9177
F02	Il5	3.9177
F01	Il4	3.8106
E09	Il23a	3.7581
C08	Cxcl16	3.7321
C06	Cxcl12	3.7064
G10	Tnfsf13b	3.4581
A10	Ccl19	3.1602
F03	Il6	2.9897
F09	Mif	2.6027
C10	Cxcl5	2.4284
D04	Ifng	2.2815
B05	Ccl5	2.042
A12	Ccl20	−2.0705
D06	Il11	−2.3134
D11	Il16	−2.5315
E11	Il27	−2.8284
B11	Csf2	−2.9079
A03	Bmp4	−3.0738
G02	Ppbp	−3.2043
C09	Cxcl3	−3.6808
G05	Thpo	−3.9177
D02	Hc	−4.4076
E03	Il1a	−8.9383
A04	Bmp6	−14.42

### Over-expression of miR-155 induces cytokine changes in human breast cell lines

**Figure 4:**
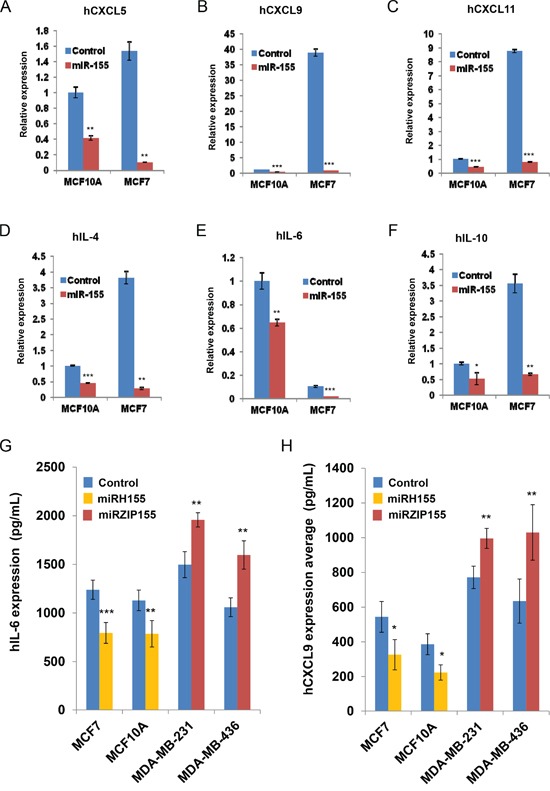
miR-155 dependent cytokine/chemokine regulation in human breast cancer cells **A-F.** Real-time PCR results of chemokines and cytokines from the human breast cells with miR-155 mimic (in miR-155- low MCF10A or MCF7) or lentivirus mediated miR-155 knockdown (in miR-155 high MDA-MB-231 or MDA-MB-436). **G, H.** ELISA results of culture supernatant from the indicated cancer cells infected with miR-155 over-expression (miRH155) or miR-155 knockdown (miRZIP155) lentiviruses. *p<0.05, **p<0.01, ***p<0.001 for all panels. The experiments were performed three times, and the representative result is shown.

We next examined if miR-155-mediated cytokine/chemokine regulation also occurs in human breast cancer cells. We transfected MCF7 and MCF10A cells that have low levels of endogenous miR-155 with miR-155 mimic. The transfectants were then indirectly co-cultured with differentiated adipocytes as shown in Figure 3B. We measured RNA expression levels of cytokine/chemokines including CXCL5, 9,11 and IL-4,6,10, which are shown to be up-regulated in Figure 3C-H. As shown in Figure 4A-F, over-expression of miR-155 mimic in both cells dramatically decreased the expression of the cytokines, although the relative basal level was low for some cytokines (*e.g.*, CXCL9 in MCF10A). The cytokine regulation by miR-155 was further confirmed by ELISA assay for IL-6 and CXCL9 by over expressing miR-155 in MCF7 and MCF10A (low endogenous miR-155) and silencing miR-155 in MDA-MB-231 and MDA-MB-436 (high endogenous miR-155). We observed significant reduction in the levels of the two cytokines by miR-155 over-expression (miRH155) whereas inhibition of the miR-155 (miRZIP155) increased cytokine production (Figures 4G and H). These results support our findings from the mouse model and demonstrate that the miR-155-mediated cytokine regulation occurs in human cells as well.

### C/EBP-β is responsible for the up-regulation of cytokines/chemokines

**Figure 5:**
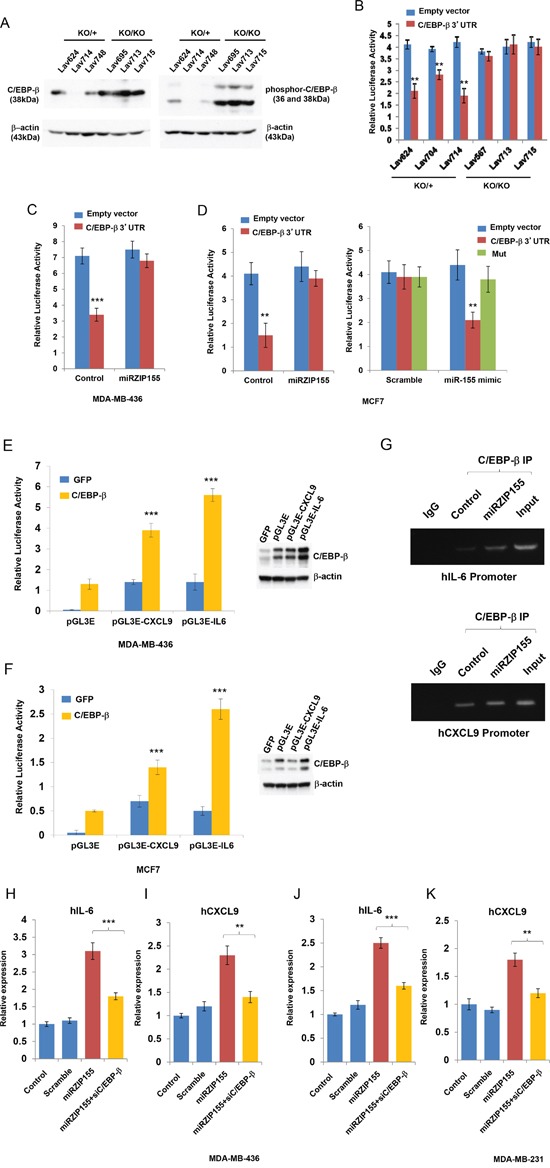
C/EBP-β is regulated by miR-155 in breast cancer cell and mediates chemokine/cytokine expression **A.** Western blot analysis of C/EBP-β and phospho-C/EBP-β in breast cancer cells from miR-155^ko/+^ (KO/+) or miR-155^ko/ko^ (KO/KO) mice. Lav624,714, and 748 cells are from KO/+, and Lav695, 713 and 715 cells are from KO/KO tumors. Size markers (in kDa) are indicated. **B.** C/EBP-β UTR reporter assay in miR-155 KO/+ or KO/KO breast cancer cells. **C, D.** C/EBP-β UTR reporter assay in MDA-MB-436 and MCF7 cells. Empty or C/EBP-β 3′UTR luciferase reporter vector (Wt) was cotransfected into MDA-MB-436 control or miRZIP155 cells (C) and MCF7 control or MCF7 miRH155 cells (D, left panel). The pMiR-C/EBP-β-3′UTR (Wt or Mut) was co-transfected with miR-155 mimic or scramble miRNA into MCF7 cells (D, right panel) and reporter assay is performed. **E, F.** Promoter reporter assays of CXCL9 and IL-6 in MDA-MB-436 (E) and MCF7 (F) cells. pGL3E is the vector only control. CEB/P-b over-expression was shown on the right panel (western blot). **G.** ChIP analysis of C/EBP-β on CXCL9 and IL-6 promoters. **H-K.** C/EBP-β knockdown abolish miR-155 mediated IL-6 and CXCL9 up-regulation in two breast cancer cell lines, MDA-MB-436 (H, I) or MDA-MB-231 cells. Size markers (in base pairs) are indicated (J, K). **p<0.01, ***p<0.001 for all panels. The experiments were performed three times, and the representative result is shown.

To understand how the absence or inhibition of miR-155 induces a set of cytokines/chemokines, we examined the possibility that miR-155 could bind to the UTR of the cytokine/chemokine genes. However, analysis using TargetScan and PicTar prediction tool resulted in no miR-155 binding sites in the UTR of these genes (data not shown). Therefore, we hypothesized that a transcription factor regulating the genes encoding these cytokine/chemokines might be targeted by miR-155. We focused on CCAAT Enhancer Binding Protein-beta (C/EBP-β) because a number of reports have shown that it regulates the expression of various kinds of cytokines in immune as well as cancer cells [27, 28]. Moreover, it is known as a direct target of miR-155 in macrophage [29]. To elucidate a possible role of C/EBP-β in breast cancer cells, we first analyzed *miR-155^+/ko^* and *miR-155^ko/ko^* breast cancer cells to measure the C/EBP-β protein levels. We found a marked increase in C/EBP-β and its phosphoylated form in *miR-155^ko/ko^* cells relative to *miR-155^+/ko^* cells (Figure 5A). In addition, we observed up-regulated luciferase activity in *miR-155^ko/ko^* cells when C/EBP-β 3′UTR reporter plasmids were introduced into three independent *miR-155^ko/+^* or *miR-155^ko/ko^* cell lines (Figure 5B). The regulation of C/EBP-β by miR-155 is further confirmed by a C/EBP-β UTR reporter assay in MDA-MB436 and MDA-MB-436-miRZIP155 (miR-155 knock-down cells) or in MCF7/MCF7-miRZIP155 cells, showing miR-155 dependent UTR regulation (Figure 5C and 5D left panel). In addition, we showed that ‘miR-155 mimic significantly inhibited the luciferase activity of the CEB/P-β 3′UTR reporter but did not affect CEB/P-β 3′UTR reporter with mutated miR-155 binding sites (Mut). Collectively, these results indicate that CEB/P-β is a direct target of miR-155.

We next searched the promoter regions of the genes encoding these cytokines/chemokines for C/EBP-β binding by using ENCODE database and found ChIP signal on IL-1β, 6, 10, 13 and CXCL9 (Supplementary Figure 4A-E). We further examined IL-6 and CXCL9, as IL-6 is a well-known target whereas the CXCL9 is uncharacterized target of the C/EBP-β in breast cancer[30, 31]. By using luciferase-based reporter constructs for these two promoters we observed that these are activated when C/EBP-β is ectopically expressed (Figure 5E and 5F). We examined direct binding of C/EBP-β to IL-6 and CXCL9 promoters by performing chromatin immunoprecipitation (ChIP, Figure 5G, Supplementary Figure 4F for quantitation). We observed a clear ChIP signal in all two promoters with marked increase upon the inhibition of miR-155 (miRZIP155) in IL-6 and CXCL9 promoters. These results suggest that C/EBP-β directly transactivates these cytokine genes. Finally, we confirmed that the up-regulation of IL-6 and CXCL9 expression mediated by miR-155 inhibition is dependent on C/EBP-β. We knocked down C/EBP-β by siRNA treatment, which reduced IL-6 and CXCL9 expression induced by miR-155 inhibition in two breast cancer cell lines (Figure 5H-5K). Taken together, these results demonstrate that C/EBP-β is, at least in part, responsible for the up-regulation of cytokine/chemokines induced by miR-155 inhibition.

### Opposing roles of miR-155 inactivation in cancer cells and the tumor microenvironment on tumor growth

**Figure 6:**
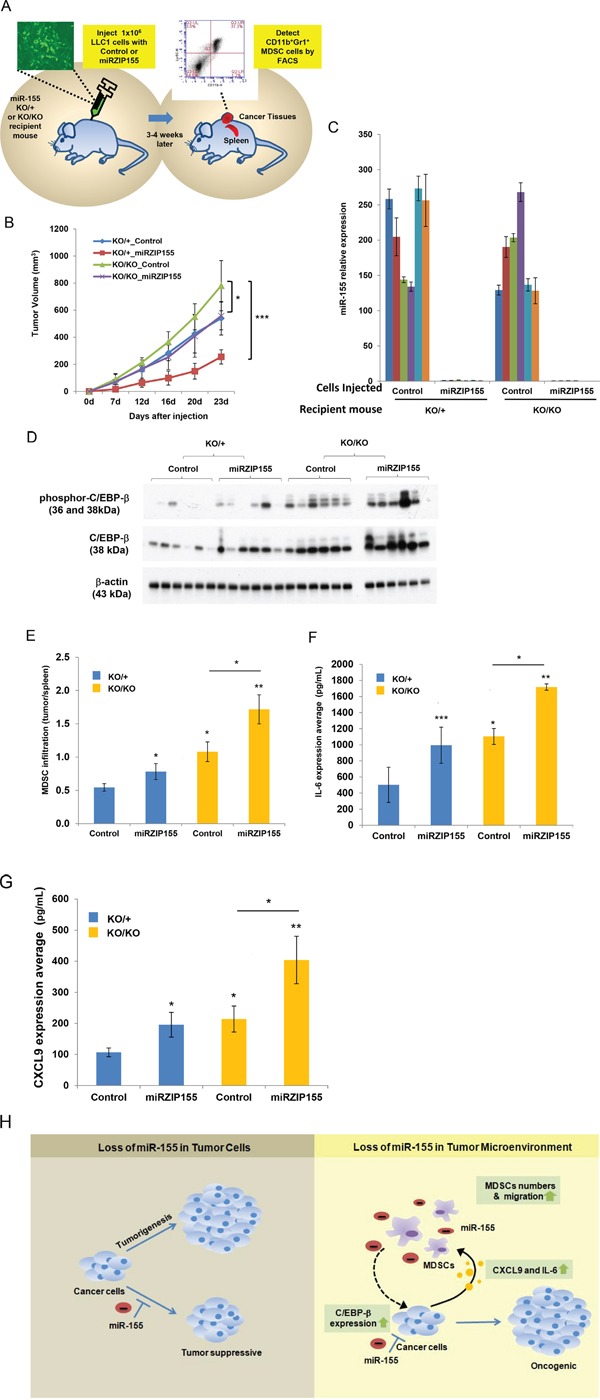
Growth defect of miR-155-knockdown cells are restored in miR-155ko/ko recipient mice **A.** Schematic representation of the experimental procedure. LLC1 and LLC1-miR-155 knockdown (miRZIP155) pair cells were introduced into either miR-155ko/+ (KO/+) or miR-155ko/ko (KO/KO) recipient mice. Tumor growth was measured and tumors were harvested at the end of the experiment. Infiltrated MDSC and molecular targets were measured by FACS or ELISA. **B.** Growth curve of each xenograft group (n=6 for each group). LLC1 control cells in KO/+ (Blue), KO/KO (green) mice; LLC1-miRZIP155 cells in KO/+ (Red), KO/KO (purple) mice. **C.** The level of miR-155 in the xenograft tumors harvested at the end. **D.** Western blot analysis of C/EBP-β and phospho-C/EBP-β levels in xenograft tumors. β-actin is used as loading control. Size markers (in kDa) are indicated. **E.** MDSC infiltration ratio in each group of the xenografts (n=6). As shown in Figure 2, the MDSC infiltration ratio is calculated by the portion of MDSC in tumor divided by the portion in spleen. **F, G.** Level of IL-6 (F) and CXCL9 (G) measured in xenograft tumors by ELISA. H. Schematic diagram of the dual roles of miR-155 in tumor and tumor microenvironment. *p<0.05, **p<0.01, ***p<0.001 for all panels. The experiments were performed twice (B) or three (C-D) times, and the representative results are shown.

The results described above strongly suggest a tumor suppressive role of miR-155 in the tumor microenvironment via C/EBP-β mediated cytokine production and MDSC infiltration. In contrast, previous findings have demonstrated an oncogenic role of miR-155 in tumor cells [32, 33]. Therefore, we hypothesized that these two opposing roles might compensate each other *in vivo*, when the miR-155 is lost in both compartments. This idea is supported by the observation that germ-line inactivation of miR-155 did not affect tumor formation in *Brca1^cko/cko^;Trp53^cko/cko^;K14 Cre*;*miR-155^ko/ko^* mice (Figure 1A). To test this hypothesis, we generated Lewis Lung carcinoma cells (LLC1) with stable knockdown of miR-155 and injected the cells into isogenic (C57BL/6) *miR-155^ko/+^* or *miR-155^ko/ko^* mice and monitored tumor growth (Figure 6A and B, Supplementary Figure 5A). The level of miR-155 in tumors was confirmed by real time PCR (Figure 6C). We observed that when injected into *miR-155^ko/+^* mice, the knockdown of miR-155 (indicated as miRZIP155) in LLC1 cells slowed down tumor growth compared to the control vector (Figure 6B; compare red line with blue line). More importantly, the growth defect of miRZIP155 LLC1 cells was restored when they were introduced into the *miR-155^ko/ko^* mice (Figure 6B, compare purple line with red line). Interestingly, the growth of miRZIP155 LLC1 cells in *miR-155^ko/ko^* mice was comparable to the growth of control LLC1 cells in *miR-155^ko/+^* mice (Figure 6B, compare blue and purple lines). These observations provide strong evidence to suggest a compensatory effect of miR-155 in the tumor and the tumor microenvironment, which may explain why we did not observe any significant difference in the tumor growth in *Brca1^cko/cko^;Trp53^cko/cko^;K14 Cre* mice in *miR-155^ko/+^* and *miR-155^ko/ko^* backgrounds (Figure 1A).

### Increased cytokine expression and MDSC infiltration is caused by the loss of miR-155 in both cancer cell and tumor microenvironment

In order to confirm our results that miR-155 dependent C/EBP-β up-regulation in cancer cells mediates cytokine up-regulation and MDSC infiltration (Figure 3, Figure 5), we analyzed the LLC1 xenograft model in more detail. First, we detected increased C/EBP-β and phospho-C/EBP-β levels in miR-155 KD LLC1 cells (Figure 6D, Supplementary Figure 5B for confirmation and 5C for quantitation). Of note, the level of C/EBP-β is increased not only by the miR-155 inhibition, but also by grafting cells into the *miR-155^ko/ko^* mice, suggesting that there are host-driven factors that induce C/EBP-β. When we measured MDSCs from the dissected tumors by FACS, we found the inhibition of miR-155 in tumor cells increase MDSC infiltration ratio (Figure 6E Blue and Red bars, Supplementary Figure 5D and E, Supplementary Table 2). This increase is further enhanced when the cells are introduced into *miR-155^ko/ko^* mice (compare red with purple bars in Figure 6E). These data suggest that loss of miR-155 in the tumor as well as in the tumor microenvironment contribute to the increased MDSC infiltration into the tumor. Furthermore, we found this increase of MDSC infiltration is correlated with the up-regulated level of IL-6 and CXCL9, as measured by ELISA (Figure 6F and G, Supplementary Figure 6A and B). Taken together, these results demonstrate a novel function of miR-155 in the tumor and its microenvironment, which is mediated by C/EBP-β and its regulatory function on cytokine expression that in turn results in altered MDSC infiltration into the tumor (Summarized in Figure 6H).

## DISCUSSION

In the past few years several studies have been published describing the role of mir-155 in tumor progression that suggest its dual role as an oncogene as well as a tumor suppressor [32–34]. However, some of the findings are conflicting. The first study published by Huffaker *et. al.* showed that the loss of miR-155 in host animal results in faster xenograft growth due to increased response of T cells to interferon gamma [13]. On the other hand, Li *et. al.* demonstrated that miR-155 promotes expansion of functional MDSCs suggesting that loss of miR-155 might negatively affect MDSC proliferation [16]. However, another recent report by Wang *et.al.* revealed that miR-155 deficiency enhances the recruitment and functions of MDSCs [17], which is beneficial to the tumor. This finding is contradictory to another study reported by Chen *et. al.* where the loss of miR-155 enhanced antitumor T cell activity and reduced MDSC infiltration into the tumor [18].

At present, it is unclear why such conflicting results have been obtained. However, these studies have all used different mouse models, either xenograft models using different cell lines or a chemically induced, acute tumor model, which may contribute to some of the variability. In this report, we analyzed a *Brca1/Trp53-*based spontaneous breast cancer model with genetic loss of miR-155. Some of our results support the notion that loss of miR-155 in MDSC is beneficial for tumor growth. Our results show that loss of miR-155 in the host animal allows more MDSCs to infiltrate into the tumor (Figure 6E, compare 1^st^ and 3^rd^, 2^nd^ and 4^th^ bar). More importantly, our findings reveal that the miR-155 deficient tumor cells also contribute to the MDSC infiltration (Figure 3A and Figure 6E; compare 1^st^ and 2^nd^, 3^rd^ and 4^th^ bar). Therefore, it is likely that MDSC infiltration is maximized when both of the tumor and MDSCs lack miR-155.

Among the likely genes that are transcriptionally affected by the co-culture system, we focused on the cytokines and chemokines because these can potentially mediate the recruitment of the MDSCs. We hypothesized that certain tumor cell driven factor can be differentially expressed in *miR-155^ko/ko^* cells that can aid in the recruitment of more MDSCs, which in turn will create a favorable microenvironment for tumor cell growth. Indeed, we showed that IL-6 and CXCL9 are such cytokine and chemokine. Future studies using neutralizing antibody against IL-6 and CXCL9 will reveal the clinical applicability of this finding.

Finally, because miR-155 is an important therapeutic target, our findings suggest that its systemic inhibition as an antitumor therapy can have detrimental effects due to increased MDSC infiltration. Even though we show only MDSC is significantly enriched in miR-155-deficient tumor microenvironment in the breast cancer model, we expect other immune effector cells to also differentially infiltrate in human cancer when miR-155 is inactivated by therapeutic agents, such as chemically modified AntagomiR. Finding such cells and understanding how the elegant interplay among these immune cells can affect tumor growth will be critical for developing better therapeutic strategies to target miR-155.

## MATERIALS AND METHODS

### Generation and genotyping of mice

*Brca1^cko/cko^;Trp53^cko/cko^;K14-Cre* mice were generated by mating *Brca1^cko/cko^* mice with *Trp53^cko/cko^* and K14-Cre mouse (all mouse lines were obtained from NCI-Frederick repository). The genotyping was performed as described previously[35]. The mice were further breeded with *miR-155^ko/ko^* (Jackson Laboratory, Bar Harbor, USA) to obtain *Brca1^cko/cko^;Trp53^cko/cko^;K14-Cre; miR-155^ko/ko^* and *Brca1^cko/cko^;Trp53^cko/cko^;K14-Cre; miR-155^ko/+^* mice. miR-155 mutation was genotyped as described previously[9]. Primers are listed in Supplementary Table 3. All animal studies were performed by the Guideline for the Care and Use of Laboratory Animals and approved by the NCI-Frederick Animal Care and the Laboratory of Animal Research in Asan Institute of Life Sciences.

### Tumor immunohistochemical and immunofluoresence staining

For histological analysis, FFPE sections were stained with hematoxylin and eosin (H&E). For immunofluoresence staining, frozen tissue sections were co-immunostained with anti-mouse Gr1-FITC (1:1000; Sigma) and anti-CD11b-Cy3 (1:1000; Sigma) antibodies. Images were captured using a LMS510 confocal microscope (ZEISS, Germany), analyzed using Image-Pro Plus 6.0 package (Media Cybernetics, MD, USA).

### FACS analysis of immune cells in spleen or tumor

To detect immune cells, tumors and spleens were processed for FACS analysis. Briefly, tumors were minced and digested in 5 ml of dissociation solution (RPMI1640 medium with Collagenase type l, 200 U/ml) for 1-2 hours at 37°C;. Spleens were crushed in RPMI1640 medium using cell strainer (100 mM, Falcon, NJ, USA). Digested tumors were also filtered and washed twice. Erythrocytes were lysed by RBC lysis buffer (Sigma-Aldrich, St. Louis, USA). 1 × 10^7^ processed cells were incubated with PE-conjugated Gr1 or CD8 mAb (BD Pharmigen, CA, USA) or FITC-conjugated CD11b or CD4 mAb in FACS buffer for 20 mins at RT. Samples were washed twice with FACS staining buffer, analyzed in the FACSCalibur (Figure 2) or Accuri Flow Cytometer (Figure 6 and Supplementary Figure 5, BD Biosciences). Data were analyzed using CellQuest Pro or CFlow software.

### Derivation of mouse primary breast cancer cells and cell lines

miR-155 heterozygous (*miR-155^ko/+^*) or knockout (*miR-155^ko/ko^*) primary cancer cells were isolated from tumor tissues using modified MEC (mammary epithelial cell) isolation procedure described previously[19]. Briefly, fresh tumor was minced and incubated with collagenase type I and IV in DMEM with antibiotics (Hyclone) for 6 hrs. The digested tissue was passed through strainer (40 mM pore size, Falcon) and the cell suspension was washed once. The primary cells were cultured in DMEM containing 10% FBS and antibiotics. MEF, EMT6, MCF7, MDA-MB-231, MDA-MB-436, HeLa, LLC1 (ATCC, Manassas, VA, USA) and 293TN (System Biosciences, SBI, Mountain View, CA, USA) cells were maintained in DMEM containing 10% FBS and 1% antibiotics. MCF10A (ATCC) cells were cultured in RPMI1640 medium with 10% FBS, hEGF (200 ng/ml), Hydrocortisol (10 mg/ml), transferrin (10 mg/ml) and 1% penicillin/streptomycin.

### Differentiation of 3T3-L1 cells to adipocytes

The 3T3-L1 preadipocyte cell line was provided by Prof. Young-Sup Song (University of Ulsan, college of Medicine) and maintained in DMEM/10% FBS. Adipocyte differentiation was induced by a mixture containing 10% FBS, 1 unit insulin (Eli Lilly), 0.5 mM 3-isobutyl-1-methylxanthine (Sigma), 2 mg/ml Dexamethasone (Sigma) and 0.5 mM Rosiglitazone (Cayman Chemical Company, Michigan, USA). Oil red O staining was performed at the end point of culture. Indirect co-culture was performed as described previously with some modifications[36]. The 3T3-L1 cells were seeded onto trans-well insert with 0.3 mm pore size (Corning Incorporated Life Sciences, MA, USA). The cells were differentiated into adipocytes as described above and then co-cultured with *miR-155^ko/+^* or *miR-155^ko/ko^* cells or cancer cell lines for 24, 48, and 72 hours.

### miR-155 knockdown or overexpression

For knockdown of miR-155, lentiviral constructs containing anti-miR-155 sequence (miRZIP155) was used. The lentiviral production was performed following the manufacturer's instructions. Control or miRZIP155-containing the lentivirus particles were infected into MDA-MB-436, MDA-MB-231 and three mouse *miR-155^ko/+^* primary cells. After 24 hours, cells were selected by Puromycin (1.5 mg/ml; Sigma) for 7 days. For miR-155 overexpression, the MCF7 and MCF10A cells were transfected with 30 nM miR-155 mimic or scrambled miRNA by G-fectin (Genolution, Seoul, Republic of Korea)

### RT2 profiler PCR array

First strand cDNA for qPCR was synthesized using the RT2 First Strand cDNA Kit (SABiosciences, MD, USA). Gene expression analyses were done using RT2 Profiler PCR Array qPCR kit with the RT2 SYBR Green qPCR Master Mix (SABiosciences), in the LightCycler 480 ll (Roche Applied Sciences, IN, USA). Expression analysis was using the manufacturer's analysis tool and gene expression was normalized to housekeeping genes. Differential expression was measured as fold expression relative to the *miR-155^ko/ko^* cells.

### Reporter construction and luciferase assay

For reporter construction, IL-6 and CXCL9 promoter regions were amplified (primer sequences in Supplementary Table 3) and introduced into the pGL3 Enhancer vector (Promega) via *Spe*I and *Hin*dIII sites. MCF7 and MDA-MB-436 cells were co-transfected with pcDNA-C/EBP-β (a gift from Dr. Young-Sup Song) and reporter vectors (pGL3-IL-6 or pGL3-CXCL9 or Empty vector). After 48 hours, luciferase activity was measured using Dual-Luciferase Reporter Assay System (Promega Corp, WI, USA) and Victor Luminometer (Perkin-Elmer, MA, USA). For C/EBP-β UTR reporter assay, sequences of the 3′ UTR for mouse and human C/EBP-β gene (NM_009883 and NM_005194) were cloned (primer sequences are in Supplementary Table 3) into the pMIR-REPORT Luciferase vector (Applied Biosystems). Mutant reporter was generated by site-directed mutagenesis within the miR-155 seed match region. The miR-155 target site in the C/EBP-β 3′ UTR was mutated by deleting the 8 nt miR-155 seed match sequence (AGCAUUAA at nucleotide positions 554–561 in the C/EBP-β 3′ UTR) using the QuikChange Site-Directed Mutagenesis kit according to manufacturer's instructions (Stratagene, CA, USA). The pMiR-C/EBP-β-3′UTR (Wt or Mut) was co-transfected into MCF7 cells either with scramble miRNA or miR-155 mimic using Lipofectamine2000 (Invitrogen, CA, USA) according to the transfection procedures.

### Chromatin immunoprecipitation (ChIP) assay

ChIP was performed by a protocol (http://cshprotocols.cshlp.org/content/2009/9/pdbprot5279. full) with minor modifications. Briefly, MDA-MB-436 cells infected with control or miRZIP155 lentiviral vectors were cross-linked with 1% formaldehyde for 10 minutes at RT. Then the cells were collected, lysed, and the chromatin was sonicated, and incubated with a C/EBP-β antibody overnight. PCR was used to detect ChIP signal with the primers listed in Supplementary Table 3.

### LLC1 Xenograft experiment

To establish subcutaneous tumors in *miR-155^ko/+^* or *miR-155^ko/ko^* mice, 1 × 10^6^ LLC1 cells infected with control or miRZIP155 lentiviruses were implanted subcutaneously. Tumor growth was measured by caliper every 3-4 days. Tumor volume was determined by the formula: length X width^2^/2.

### Enzyme-linked immunosorbent assay (ELISA)

The level of IL-6 and CXCL9 in tumor or culture supernatant was assessed by ELISA. Cell extracts were prepared from snap-frozen tumor and spleen tissues. Culture supernatants were collected from MCF10A, MCF7, MDA-MB-231 and MDA-MB-436 after 48 hrs of the indirect co-culture. The IL-6 and CXCL9 concentration in the media and tissue extracts was determined by ELISA kits from R&D Systems (Minneapolis, MN, USA). An aliquot of 100 μl of conditioned medium or 25 μl of tissue extract made to 100 μl with diluent (0.1% bovine serum albumin, 0.05% Tween 20, 10 mM Tris and 150 mM sodium chloride, pH 7.3) was analyzed.

### Western blot analysis

Western blot analysis was performed as previously described[19]. Briefly, cells were lysed in lysis buffer [150 mM NaCl, 1 % Triton X-100, 1% Sodium deoxycholate, 50 mM Tris-HCl (pH 7.5), 2 mM EDTA (pH 8.0), and 0.1% SDS]. 10∼50 μg of protein were separated on SDS PAGE, transferred to Nitrocellulose membrane, probed with anti-C/EBP-β or anti-phospho-C/EBP-β (Thr235)(1:1000, Cell Signaling) antibodies. Membranes were stripped and re-probed with anti β-actin antibody (1:1,000; Santa Cruz Biotechnology, CA, USA) to ensure equal loading. Densitometric analysis was performed using the imageJ 1.47v system.

### Quantitative RT-PCR (qRT-PCR)

The expression levels of cytokines, chemokines, and C/EBP-β were measured by SYBR Green PCR Kit, in LightCycler 480 II (Roche). The primer sequences are shown in Supplementary Table 3. Human and mouse *RpL13a* was used as an internal control gene. Relative quantification was calculated using the 2-(ΔΔCt) method [37]. For miR-155 quantization, miScript ll RT Kit (Qiagen, Hilden, Germany) and miScript SYBR Green PCR Kit (Qiagen) were used. Small nuclear RNA molecule U6 (RNU6) was used as an internal control.

### Statistics

All data are presented as means ±standard error mean (SEM). Statistical significance between two groups was calculated using *t*-test. P-value less than 0.05 was considered to be significant.

## SUPPLEMENTARY FIGURES AND TABLES






