# Supplementary material for: Loss of oncogenic miR-155 in tumor cells promotes tumor growth by enhancing C/EBP-β-mediated MDSC infiltration
Source: Oncotarget. 2016 Feb 3;7(10):11094–112. doi: 10.18632/oncotarget.7150 (PMC4905460; doi:10.18632/oncotarget.7150)
Supplement: Supplementary file 1 [file oncotarget-07-11094-s001.pdf]

SUPPLEMENTRAY FIGURES AND TABLES

Lav 539 (*$Brca1^{cko/cko}$;$Trp53^{cko/cko}$;K14 Cre;***$miR\text{-}155^{ko/+}$**)

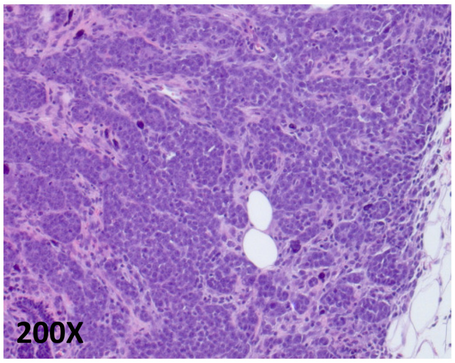

Lav 466 (*$Brca1^{cko/cko}$;$Trp53^{cko/cko}$;K14 Cre;***$miR\text{-}155^{ko/ko}$**)

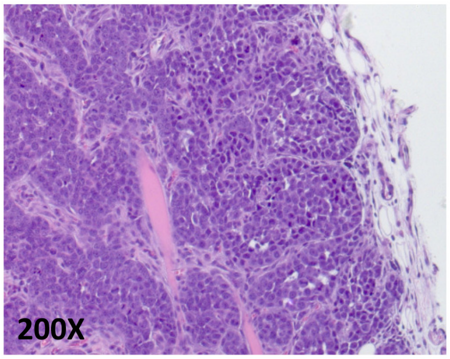

Lav 543 (*$Brca1^{cko/cko}$;$Trp53^{cko/cko}$;K14 Cre;***$miR\text{-}155^{ko/+}$**)

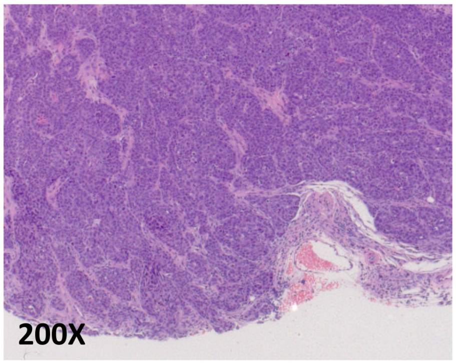

Lav 445 (*$Brca1^{cko/cko}$;$Trp53^{cko/cko}$;K14 Cre;***$miR\text{-}155^{ko/ko}$**)

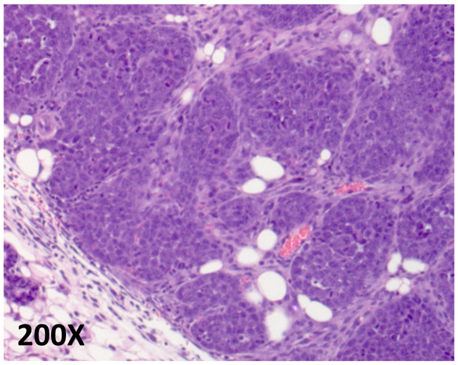

Supplementary Figure S1: Representative pictures of H&E staining of the tumor dissected from the *$Brca1^{cko/cko}$;$Trp53^{cko/cko}$; K14 Cre;$miR\text{-}155^{ko/+}$ (left) or $Brca1^{cko/cko}$;$Trp53^{cko/cko}$;K14 Cre;$miR\text{-}155^{ko/ko}$(right)* mice. Tumors from both genotypic groups show very similar histology. Notice similar amount of stroma separating the packets of neoplastic cells.

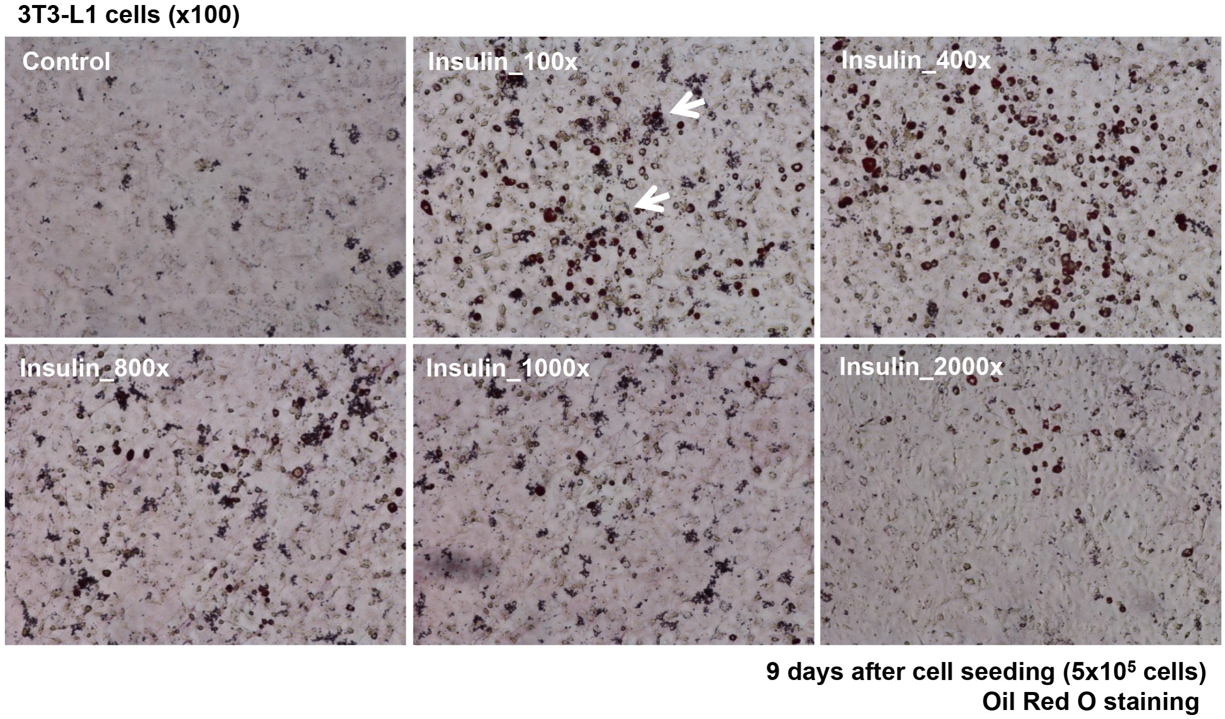

Supplementary Figure S2: Oil red O staining pictures of adipocytes differentiated from 3T3-L1 cells. Cells were treated with different concentration of insulin to find an optimal condition for adipogenesis, and then stained at 9 days after cell seeding.

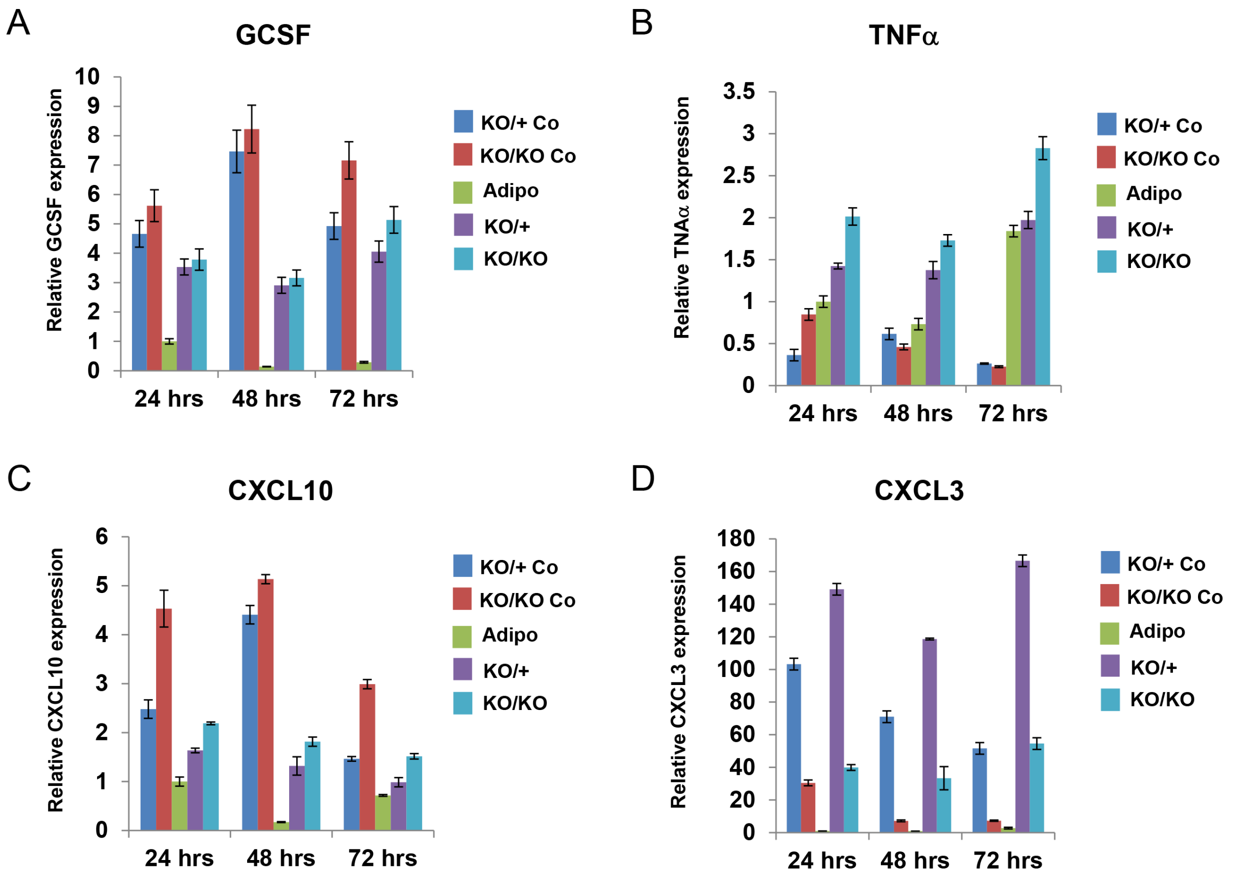

Supplementary Figure S3: (A-D) Real time PCR results of GCSF, TNFα, CXCL10 and CXCL3 at 24, 48, and 72 hours after co-culture of *miR-155*$^{ko/+}$ (KO/+) or *miR-155*$^{ko/ko}$ (KO/KO) cells with adipocytes-differentiated 3T3-L1 cells. Representative result of three experiments.

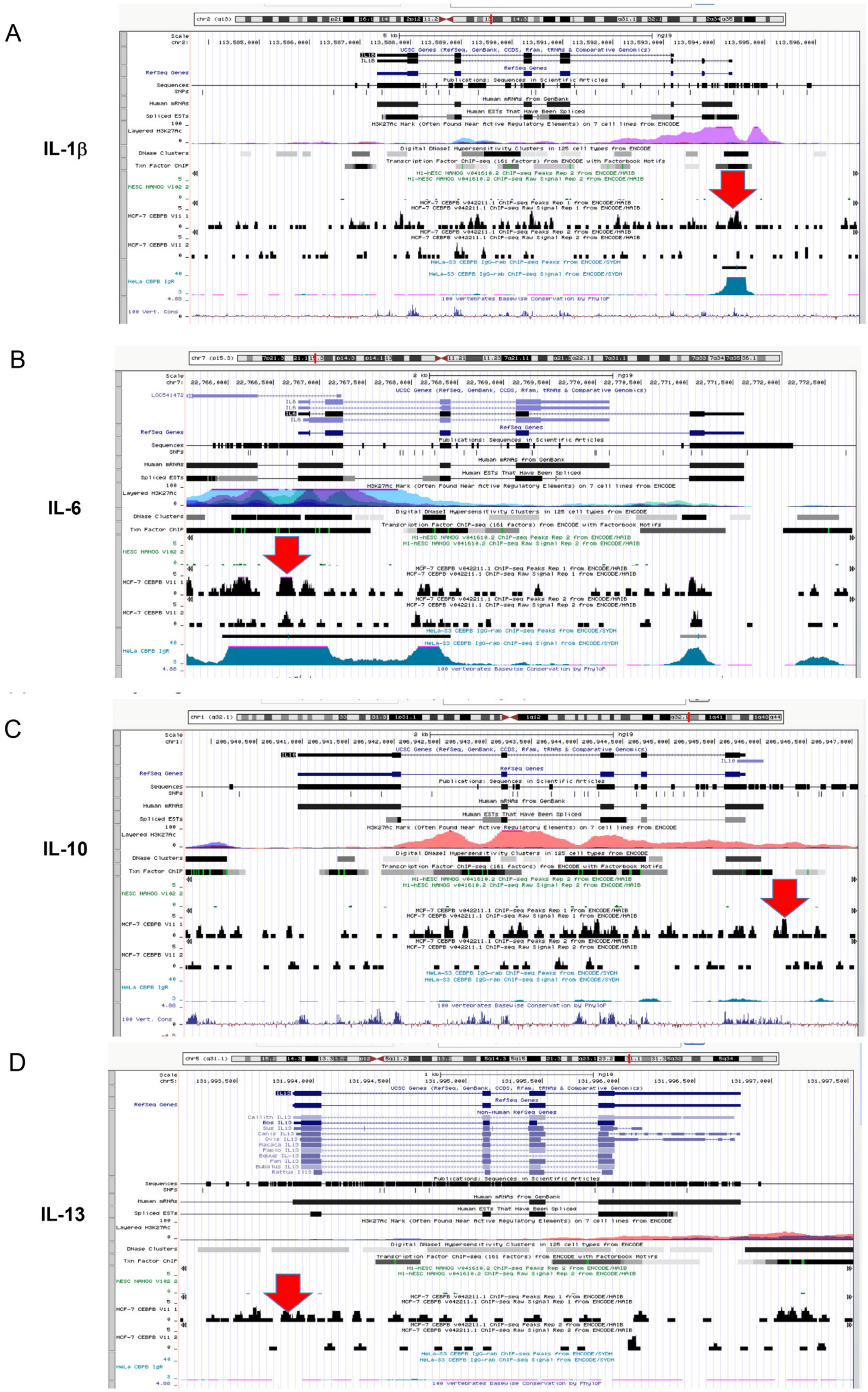

(*Continued*)

E

CXCL9

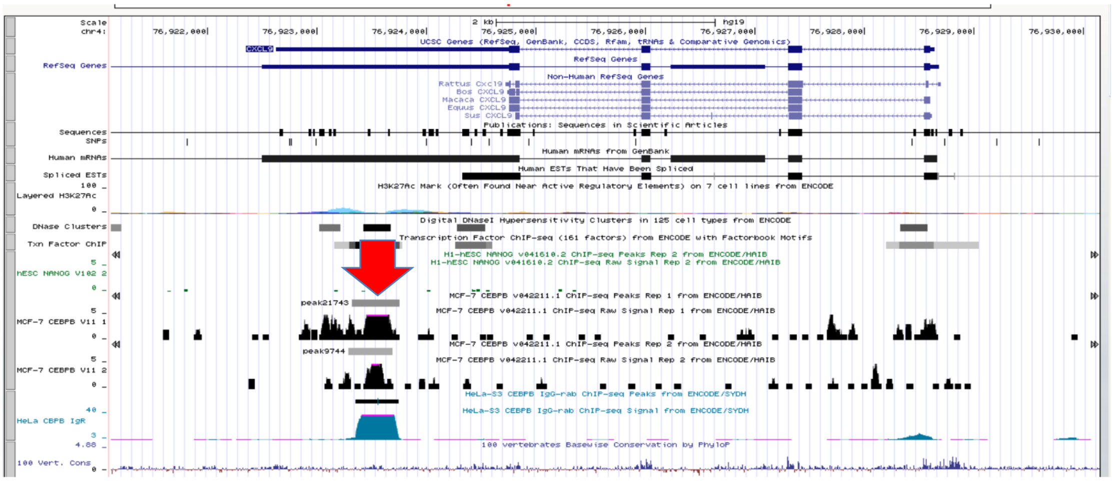

F

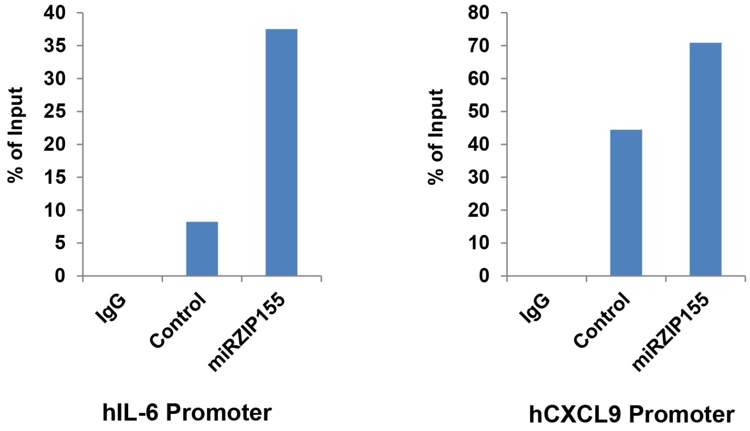

Supplementary Figure S4: Snapshot pictures of the ENCODE data presented in UCSC genome browser. A-E. The promoter regions of the genes encoding IL-1β, 6, 10, 13 and CXCL9 were searched for C/EBP-β binding sites and putative binding sites are indicated by red arrow. **F.** Densitometry results obtained from ChIP assays of C/EBP-β on CXCL9 (right) and IL-6 promoters (left).

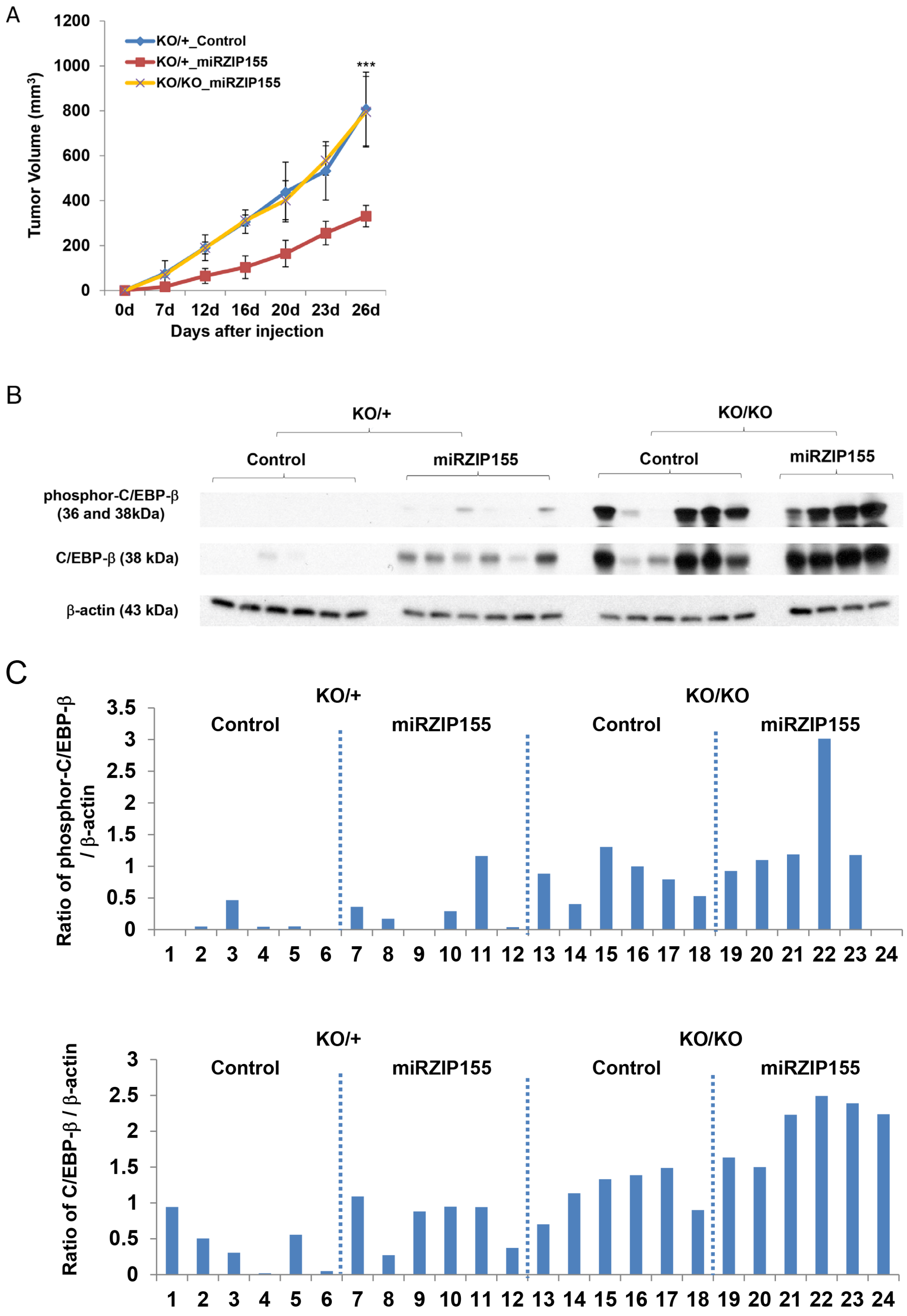
A
1200
1000
800
600
400
200
0
Tumor Volume (mm3)
KO/+_Control
KO/+_miRZIP155
KO/KO_miRZIP155
***
0d 7d 12d 16d 20d 23d 26d
Days after injection
B
KO/+
KO/KO
Control
miRZIP155
Control
miRZIP155
phosphor-C/EBP-β
(36 and 38kDa)
C/EBP-β (38 kDa)
β-actin (43 kDa)
C
KO/+
KO/KO
Control
miRZIP155
Control
miRZIP155
Ratio of phosphor-C/EBP-β / β-actin
3.5
3
2.5
2
1.5
1
0.5
0
1 2 3 4 5 6 7 8 9 10 11 12 13 14 15 16 17 18 19 20 21 22 23 24
KO/+
KO/KO
Control
miRZIP155
Control
miRZIP155
Ratio of C/EBP-β / β-actin
3
2.5
2
1.5
1
0.5
0
1 2 3 4 5 6 7 8 9 10 11 12 13 14 15 16 17 18 19 20 21 22 23 24

(Continued)

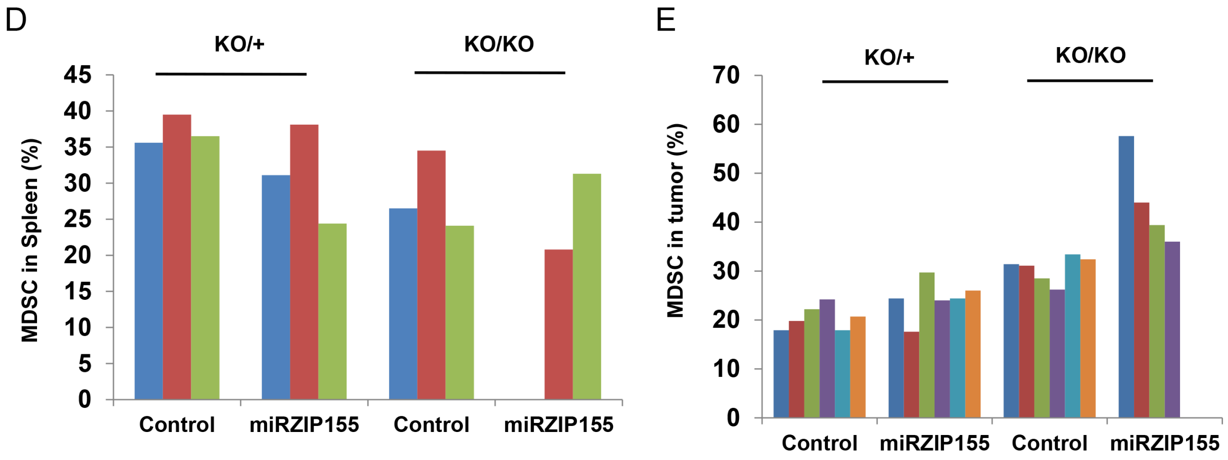

Supplementary Figure S5: *In vivo* effect of miR-155 loss in cancer cells or microenvironment. A. Growth curve of xenografts (n=4 or 6 for each group). LLC1 control cells were injected into *miR-155*$^{ko/+}$ (KO/+) (Blue), *miR-155*$^{ko/ko}$ (KO/KO) (red) mouse; LLC-miR-155 KD cells were injected into miR-155 KO/+ (Yellow). **B.** The level of C/EBP-β and phospho-C/EBP-β in xenograft tumors were determined by Western blot. **C.** Densitometry results of phosphor-C/EBP-β or C/EBBP-β /β-action obtained from western data (Figure 6D). **D** and **E.** Tumor and spleen MDSC infiltration ratios in each group of the xenografts (n=4 or 6).

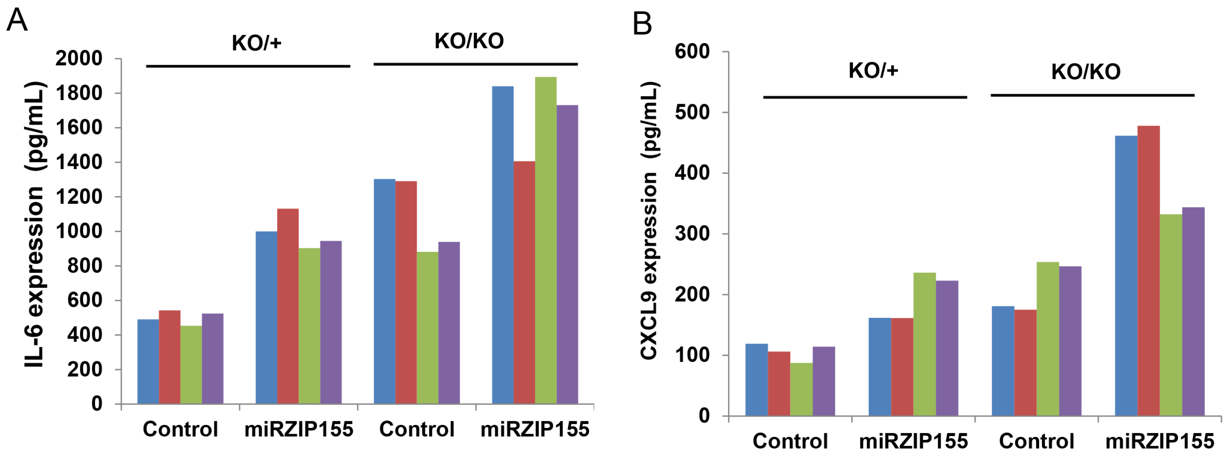

Supplementary Figure S6: Level of IL-6 A. and CXCL9 B. measured in xenograft tumors by ELISA.

Supplementary Table S1: Histological summary of the tumors harvested from *miR-155*$^{ko/+}$ (KO/+) or *miR-155*$^{ko/ko}$ (KO/KO) mice

miR-155 genotype	Animal ID	PHL#	Tumor Morphology
−/−	Lav696	1937	Carcinoma, glandular to cribriform
	Lav293	1948	Carcinoma, glandular to cribriform
	Lav697	1938	Carcinoma, glandular
	Lav653	1933	Carcinoma, glandular w foci of squamous differentiation/ keratin production
	Lav630	1932	Carcinoma, glandular
	Lav314	1926	Carcinoma, glandular
	Lav715	1940	Carcinoma, glandular
+/−	Lav734	1941	Sarcoma
	Lav624	1931	Sarcoma
	Lav622	1930	Carcinoma, glandular
	Lav670	1936	Carcinoma, glandular
	Lav707	1939	Carcinoma, glandular

Supplementary Table S2: Summary of FACS analysis of MDSCs from cancer tissues or spleens obtained from miR-155 KO/+ or KO/KO tumor bearing mouse (First (left) and second experiment (right))

See Supplementary File 1

Supplementary Table S3: List of Primers used in this study

See Supplementary File 2
