# Supplementary material for: Loss of oncogenic miR-155 in tumor cells promotes tumor growth by enhancing C/EBP-β-mediated MDSC infiltration
Source: Oncotarget. 2016 Feb 3;7(10):11094–112. doi: 10.18632/oncotarget.7150 (PMC4905460; doi:10.18632/oncotarget.7150)
Supplement: Supplementary file 2 [file oncotarget-07-11094-s002.ppt]

## Slide 1
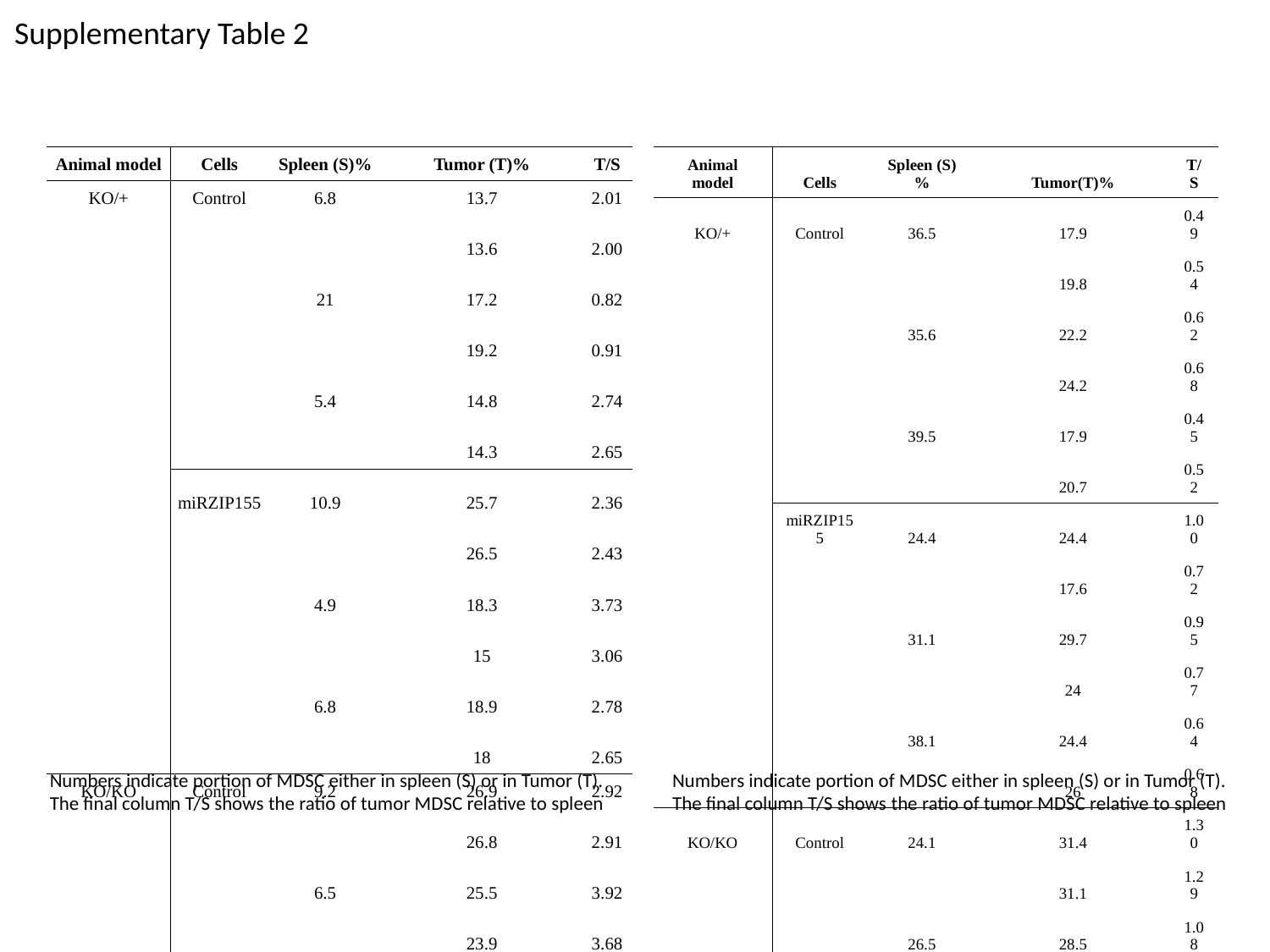

Supplementary Table 2
| Animal model | Cells | Spleen (S)% | Tumor (T)% | T/S |
| --- | --- | --- | --- | --- |
| KO/+ | Control | 6.8 | 13.7 | 2.01 |
| | | | 13.6 | 2.00 |
| | | 21 | 17.2 | 0.82 |
| | | | 19.2 | 0.91 |
| | | 5.4 | 14.8 | 2.74 |
| | | | 14.3 | 2.65 |
| | miRZIP155 | 10.9 | 25.7 | 2.36 |
| | | | 26.5 | 2.43 |
| | | 4.9 | 18.3 | 3.73 |
| | | | 15 | 3.06 |
| | | 6.8 | 18.9 | 2.78 |
| | | | 18 | 2.65 |
| KO/KO | Control | 9.2 | 26.9 | 2.92 |
| | | | 26.8 | 2.91 |
| | | 6.5 | 25.5 | 3.92 |
| | | | 23.9 | 3.68 |
| | | 4.2 | 22.1 | 5.26 |
| | | | 22.2 | 5.29 |
| | miRZIP155 | 6.9 | 41.6 | 6.03 |
| | | | 44.2 | 6.41 |
| | | 3.5 | 26.8 | 7.66 |
| | | | 26.5 | 7.57 |
| | | 2.7 | 21.7 | 8.04 |
| | | | 18 | 6.67 |
| Animal model | Cells | Spleen (S)% | Tumor(T)% | T/S |
| --- | --- | --- | --- | --- |
| KO/+ | Control | 36.5 | 17.9 | 0.49 |
| | | | 19.8 | 0.54 |
| | | 35.6 | 22.2 | 0.62 |
| | | | 24.2 | 0.68 |
| | | 39.5 | 17.9 | 0.45 |
| | | | 20.7 | 0.52 |
| | miRZIP155 | 24.4 | 24.4 | 1.00 |
| | | | 17.6 | 0.72 |
| | | 31.1 | 29.7 | 0.95 |
| | | | 24 | 0.77 |
| | | 38.1 | 24.4 | 0.64 |
| | | | 26 | 0.68 |
| KO/KO | Control | 24.1 | 31.4 | 1.30 |
| | | | 31.1 | 1.29 |
| | | 26.5 | 28.5 | 1.08 |
| | | | 26.2 | 0.99 |
| | | 34.5 | 33.4 | 0.97 |
| | | | 32.4 | 0.94 |
| | miRZIP155 | 31.3 | 57.6 | 1.84 |
| | | | 44 | 1.41 |
| | | 20.8 | 39.4 | 1.89 |
| | | | 36 | 1.73 |
Numbers indicate portion of MDSC either in spleen (S) or in Tumor (T).
The final column T/S shows the ratio of tumor MDSC relative to spleen
Numbers indicate portion of MDSC either in spleen (S) or in Tumor (T).
The final column T/S shows the ratio of tumor MDSC relative to spleen
