# Supplementary material for: Loss of oncogenic miR-155 in tumor cells promotes tumor growth by enhancing C/EBP-β-mediated MDSC infiltration
Source: Oncotarget. 2016 Feb 3;7(10):11094–112. doi: 10.18632/oncotarget.7150 (PMC4905460; doi:10.18632/oncotarget.7150)
Supplement: Supplementary file 3 [file oncotarget-07-11094-s003.pptx]

## Slide 1
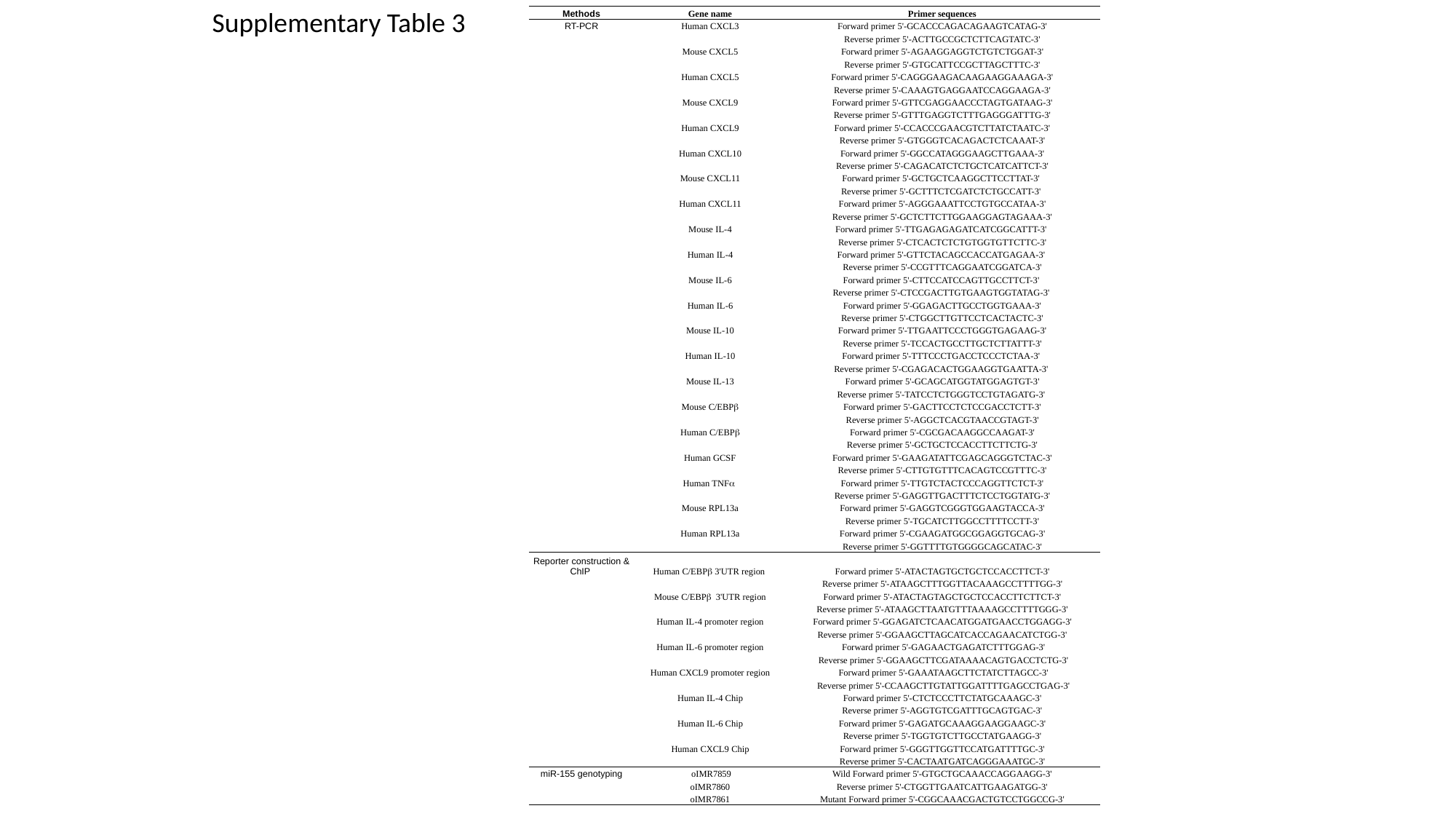

Supplementary Table 3
| Methods | Gene name | Primer sequences |
| --- | --- | --- |
| RT-PCR | Human CXCL3 | Forward primer 5'-GCACCCAGACAGAAGTCATAG-3' |
| | | Reverse primer 5'-ACTTGCCGCTCTTCAGTATC-3' |
| | Mouse CXCL5 | Forward primer 5'-AGAAGGAGGTCTGTCTGGAT-3' |
| | | Reverse primer 5'-GTGCATTCCGCTTAGCTTTC-3' |
| | Human CXCL5 | Forward primer 5'-CAGGGAAGACAAGAAGGAAAGA-3' |
| | | Reverse primer 5'-CAAAGTGAGGAATCCAGGAAGA-3' |
| | Mouse CXCL9 | Forward primer 5'-GTTCGAGGAACCCTAGTGATAAG-3' |
| | | Reverse primer 5'-GTTTGAGGTCTTTGAGGGATTTG-3' |
| | Human CXCL9 | Forward primer 5'-CCACCCGAACGTCTTATCTAATC-3' |
| | | Reverse primer 5'-GTGGGTCACAGACTCTCAAAT-3' |
| | Human CXCL10 | Forward primer 5'-GGCCATAGGGAAGCTTGAAA-3' |
| | | Reverse primer 5'-CAGACATCTCTGCTCATCATTCT-3' |
| | Mouse CXCL11 | Forward primer 5'-GCTGCTCAAGGCTTCCTTAT-3' |
| | | Reverse primer 5'-GCTTTCTCGATCTCTGCCATT-3' |
| | Human CXCL11 | Forward primer 5'-AGGGAAATTCCTGTGCCATAA-3' |
| | | Reverse primer 5'-GCTCTTCTTGGAAGGAGTAGAAA-3' |
| | Mouse IL-4 | Forward primer 5'-TTGAGAGAGATCATCGGCATTT-3' |
| | | Reverse primer 5'-CTCACTCTCTGTGGTGTTCTTC-3' |
| | Human IL-4 | Forward primer 5'-GTTCTACAGCCACCATGAGAA-3' |
| | | Reverse primer 5'-CCGTTTCAGGAATCGGATCA-3' |
| | Mouse IL-6 | Forward primer 5'-CTTCCATCCAGTTGCCTTCT-3' |
| | | Reverse primer 5'-CTCCGACTTGTGAAGTGGTATAG-3' |
| | Human IL-6 | Forward primer 5'-GGAGACTTGCCTGGTGAAA-3' |
| | | Reverse primer 5'-CTGGCTTGTTCCTCACTACTC-3' |
| | Mouse IL-10 | Forward primer 5'-TTGAATTCCCTGGGTGAGAAG-3' |
| | | Reverse primer 5'-TCCACTGCCTTGCTCTTATTT-3' |
| | Human IL-10 | Forward primer 5'-TTTCCCTGACCTCCCTCTAA-3' |
| | | Reverse primer 5'-CGAGACACTGGAAGGTGAATTA-3' |
| | Mouse IL-13 | Forward primer 5'-GCAGCATGGTATGGAGTGT-3' |
| | | Reverse primer 5'-TATCCTCTGGGTCCTGTAGATG-3' |
| | Mouse C/EBPb | Forward primer 5'-GACTTCCTCTCCGACCTCTT-3' |
| | | Reverse primer 5'-AGGCTCACGTAACCGTAGT-3' |
| | Human C/EBPb | Forward primer 5'-CGCGACAAGGCCAAGAT-3' |
| | | Reverse primer 5'-GCTGCTCCACCTTCTTCTG-3' |
| | Human GCSF | Forward primer 5'-GAAGATATTCGAGCAGGGTCTAC-3' |
| | | Reverse primer 5'-CTTGTGTTTCACAGTCCGTTTC-3' |
| | Human TNFa | Forward primer 5'-TTGTCTACTCCCAGGTTCTCT-3' |
| | | Reverse primer 5'-GAGGTTGACTTTCTCCTGGTATG-3' |
| | Mouse RPL13a | Forward primer 5'-GAGGTCGGGTGGAAGTACCA-3' |
| | | Reverse primer 5'-TGCATCTTGGCCTTTTCCTT-3' |
| | Human RPL13a | Forward primer 5'-CGAAGATGGCGGAGGTGCAG-3' |
| | | Reverse primer 5'-GGTTTTGTGGGGCAGCATAC-3' |
| Reporter construction & ChIP | Human C/EBPb 3'UTR region | Forward primer 5'-ATACTAGTGCTGCTCCACCTTCT-3' |
| | | Reverse primer 5'-ATAAGCTTTGGTTACAAAGCCTTTTGG-3' |
| | Mouse C/EBPb 3'UTR region | Forward primer 5'-ATACTAGTAGCTGCTCCACCTTCTTCT-3' |
| | | Reverse primer 5'-ATAAGCTTAATGTTTAAAAGCCTTTTGGG-3' |
| | Human IL-4 promoter region | Forward primer 5'-GGAGATCTCAACATGGATGAACCTGGAGG-3' |
| | | Reverse primer 5'-GGAAGCTTAGCATCACCAGAACATCTGG-3' |
| | Human IL-6 promoter region | Forward primer 5'-GAGAACTGAGATCTTTGGAG-3' |
| | | Reverse primer 5'-GGAAGCTTCGATAAAACAGTGACCTCTG-3' |
| | Human CXCL9 promoter region | Forward primer 5'-GAAATAAGCTTCTATCTTAGCC-3' |
| | | Reverse primer 5'-CCAAGCTTGTATTGGATTTTGAGCCTGAG-3' |
| | Human IL-4 Chip | Forward primer 5'-CTCTCCCTTCTATGCAAAGC-3' |
| | | Reverse primer 5'-AGGTGTCGATTTGCAGTGAC-3' |
| | Human IL-6 Chip | Forward primer 5'-GAGATGCAAAGGAAGGAAGC-3' |
| | | Reverse primer 5'-TGGTGTCTTGCCTATGAAGG-3' |
| | Human CXCL9 Chip | Forward primer 5'-GGGTTGGTTCCATGATTTTGC-3' |
| | | Reverse primer 5'-CACTAATGATCAGGGAAATGC-3' |
| miR-155 genotyping | oIMR7859 | Wild Forward primer 5'-GTGCTGCAAACCAGGAAGG-3' |
| | oIMR7860 | Reverse primer 5'-CTGGTTGAATCATTGAAGATGG-3' |
| | oIMR7861 | Mutant Forward primer 5'-CGGCAAACGACTGTCCTGGCCG-3' |
